# Immune Checkpoint Inhibitors: New Weapons Against Medullary Thyroid Cancer?

**DOI:** 10.3389/fendo.2021.667784

**Published:** 2021-04-14

**Authors:** Sergio Di Molfetta, Andrea Dotto, Giuseppe Fanciulli, Tullio Florio, Tiziana Feola, Annamaria Colao, Antongiulio Faggiano

**Affiliations:** ^1^ Department of Emergency and Organ Transplantation, Section of Internal Medicine, Endocrinology, Andrology and Metabolic Diseases, University of Bari Aldo Moro, Bari, Italy; ^2^ Endocrinology Unit, IRCCS Ospedale Policlinico San Martino, Genova, Italy; ^3^ Department of Internal Medicine, University of Genova, Genova, Italy; ^4^ Neuroendocrine Unit, Department of Medical, Surgical and Experimental Sciences, University of Sassari—Endocrine Unit, Azienda Ospedaliera Universitaria Sassari, Sassari, Italy; ^5^ IRCCS Ospedale Policlinico San Martino, Genova, Italy; ^6^ Department of Experimental Medicine, “Sapienza” University of Rome, Rome, Italy; ^7^ Neuroendocrinology, Neuromed Institute, IRCCS, Pozzilli, Italy; ^8^ Department of Clinical Medicine and Surgery, Endocrinology Unit, University Federico II, Naples, Italy; ^9^ Endocrinology Unit, Department of Clinical and Molecular Medicine, Sant’Andrea Hospital, Sapienza University of Rome, Rome, Italy

**Keywords:** medullary thyroid carcinoma, immune checkpoint inhibitors, avelumab, durvalumab, ipilimumab, nivolumab, pembrolizumab

## Abstract

Medullary thyroid carcinoma is a rare neuroendocrine neoplasm that originates from thyroid C cells. Surgery, with complete resection of the tumor, is the only curative approach. However, in most cases, the tumor recurs at locoregional or metastatic level. In this setting, the management remains challenging. In recent years, the immune checkpoint inhibitors have provided promise for changing the cancer treatment paradigm through the application of new approaches that enhance the body’s natural antitumor defenses. The aim of this review is to summarize and discuss available data on efficacy and safety of the Food and Drug Administration-approved immune checkpoint inhibitors in patients with medullary thyroid carcinoma. After an extensive search, we found 7 useful data sources (one single-case report, one short article with very preliminary data, five ongoing registered clinical trials). Despite the lack of published evidence regarding the use of immune check point inhibitors, it must be considered that all the ongoing registered clinical trials saw first light in the last three years, thus indicating a growing interest of researchers in this field. Results coming from these trials, and hopefully, in the next future, from additional trials, will help to clarify whether this class of drugs may represent a new weapon in favor of patients with medullary thyroid carcinoma.

## Introduction

Medullary thyroid carcinoma (MTC) is a rare neuroendocrine neoplasm (NEN) that originates from thyroid C cells.

Surgery, with complete resection of the tumor, is the only curative approach (1). However, in many patients, the tumor displays an aggressive behavior, resulting in persistence or locoregional and distant disease recurrence. In this setting, the management remains challenging (1, 2).

Tyrosine kinase inhibitors (TKIs) vandetanib and cabozantinib have shown to improve progression-free survival (PFS), and are currently available as approved agents for the treatment of progressive MTC. However, both drugs may cause grade III or IV adverse events (AEs), classified according to the Common Terminology Criteria for Adverse Events of the National Cancer Institute (3, 4).

In 2020, the new generation TKIs selpercatinib and praseltinib gained the Food and Drug Administration (FDA) approval in patients with advanced/metastatic rearranged during transfection (RET) gene-mutant MTC who require systemic therapy, therefore widening the spectrum of available therapies. However, also for these drugs severe AEs have been reported (5, 6)

Therapeutic options also include radionuclide therapy, such as peptide receptor radionuclide therapy (i.e. lutetium-177 and yttrium-90 labeled somatostatin analogs) (7), and iodine-131-metaiodobenzylguanidine (8). However, radionuclide therapy is not approved for MTC treatment.

In recent years, immunotherapy has provided promise for changing the cancer treatment paradigm through the application of new approaches that enhance the body’s natural antitumor defenses.

**Figure 1:**
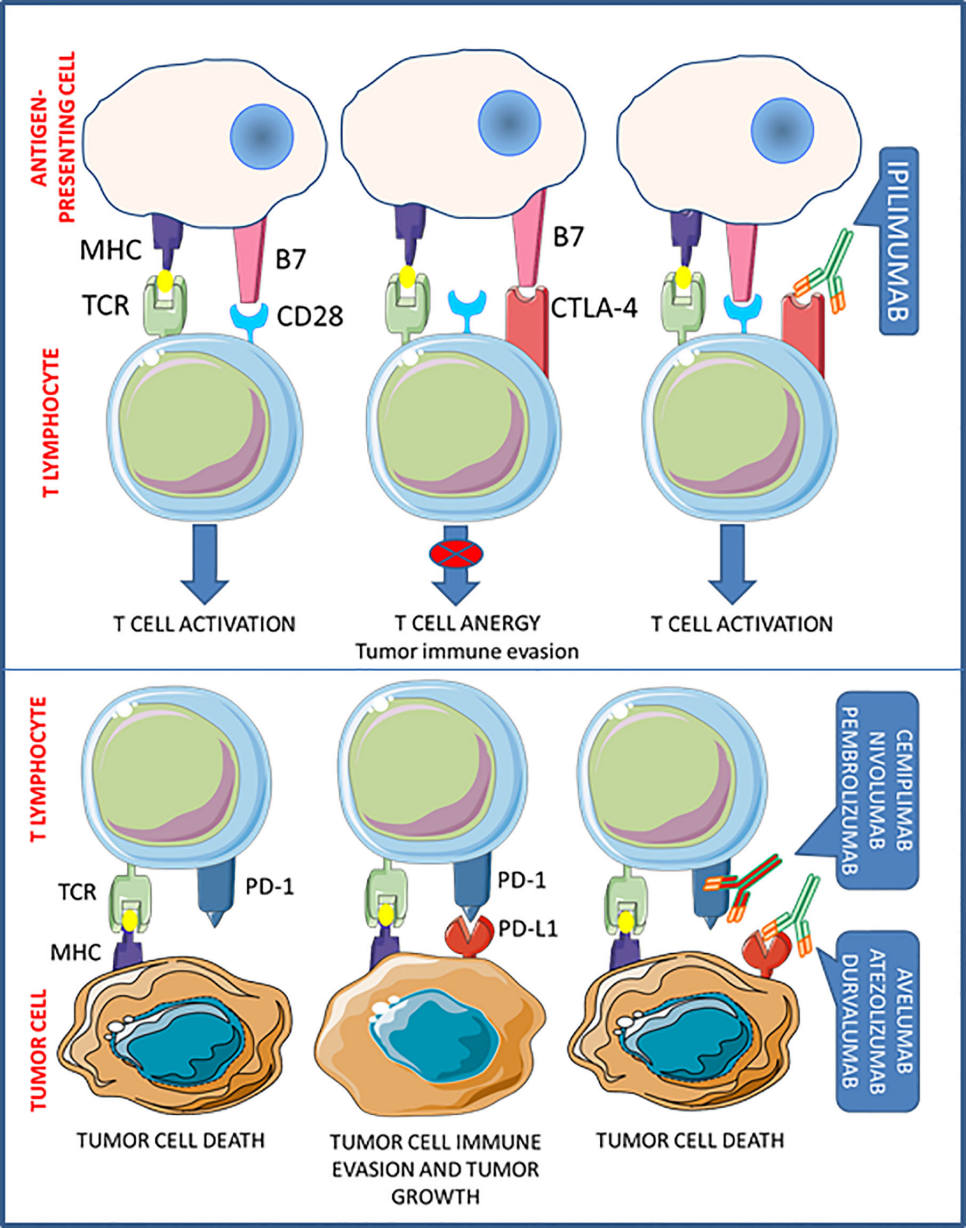
Mechanisms of action for FDA-approved immune checkpoint inhibitors.

One of the main mechanisms by which tumors escape host immune surveillance is the so-called cancer immunoediting. Acting on immune checkpoints, tumor cells promote the development of an immunosuppressive environment, to prevent the activation of T cell cytotoxicity. Thus the interfering with immune checkpoint signaling, to restore T cell functioning, is nowadays considered one of the most effective novel antitumor treatment goals (9). To date, antibodies targeted against the cytotoxic T-lymphocyte-associated protein 4 (CTLA-4) (i.e. ipilimumab), the programmed cell death protein-1 (PD-1) (i.e. cemiplimab, nivolumab, and pembrolizumab), and the programmed death-ligand-1 (PD-L1) (i.e. atezolizumab, avelumab, and durvalumab), have been approved by the FDA for human use with the aim to re-activate patient antitumor immunity (**Figure 1**). These drugs, referred as immune checkpoints inhibitors (ICIs), demonstrated significant clinical effectiveness in the treatment of advanced solid tumors and a favorable safety profile, so that entered in the standard clinical practice for several malignancies (10) (**Table 1**).

**Table 1:** FDA-approved immune checkpoint inhibitors.

Drug	Brand Name	U.S. Approval	Molecular target	Antibody Description	Indications	Most common adverse reactions
Atezolizumab	Tecentriq^®^	October 2016	PD-L1	Humanized monoclonal antibody (IgG1-kappa)	Urothelial carcinoma, NSCLC, triple-negative breast cancer	Fatigue, nausea, constipation, cough, dyspnea, and decreased appetite
Avelumab	Bavencio^®^	March 2017	PD-L1	Fully human monoclonal antibody (IgG1-lambda)	MCC, urothelial carcinoma, RCC	Fatigue, musculoskeletal pain, diarrhea, nausea, infusion-related reaction, rash, decreased appetite, peripheral edema, and urinary tract infection
Cemiplimab	Libtayo^®^	September 2018	PD-1	Fully human monoclonal antibody (IgG4-kappa)	cSCC	Fatigue, rash and diarrhea
Durvalumab	Imfinzi^®^	May 2015	PD-L1	Fully human monoclonal antibody (IgG1-kappa)	Urothelial carcinoma, NSCLC	Fatigue, musculoskeletal pain, constipation, decreased appetite, nausea, peripheral edema, urinary tract infection, cough, pneumonitis/radiation pneumonitis, upper respiratory tract infections, dyspnea, rash and alopecia
Ipilimumab	Yervoy^®^	March 2011	CTLA-4	Fully human monoclonal antibody (IgG1-kappa)	Melanoma, RCC	Fatigue, diarrhea, pruritus, rash, and colitis. Additional AR at high doses include nausea, vomiting, headache, weight loss, pyrexia, decreased appetite, and insomnia
Nivolumab	Opdivo^®^	December 2014	PD-1	Fully human monoclonal antibody (IgG4-kappa)	Melanoma, NSCLC, SCLC, RCC, cHL, HNSCC, urothelial carcinoma, MSI-H or dMMR colorectal cancer, HCC	Fatigue, rash, musculoskeletal pain, pruritus, diarrhea, nausea, asthenia, cough, dyspnea, constipation, decreased appetite, back pain, arthralgia, upper respiratory tract infection, pyrexia, headache, abdominal pain, and vomiting
Pembrolizumab	Keytruda^®^	September 2014	PD-1	Humanized monoclonal antibody (IgG4-kappa)	Melanoma, NSCLC, HNSCC, cHL, PMBCL, urothelial carcinoma, MSI-H cancer, gastric cancer, cervical cancer, HCC, MCC	Fatigue, musculoskeletal pain, decreased appetite, pruritus, diarrhea, nausea, rash, pyrexia, cough, dyspnea, constipation, pain, and abdominal pain

cHL, classical Hodgkin lymphoma; cSCC, cutaneous squamous cell carcinoma; dMRR, deficient mismatch repair; HCC, hepatocellular carcinoma; HNSCC, head and neck squamous cell carcinoma; MCC, Merkel cell carcinoma; MSI-H, high level microsatellite instability; NSCLC, non-small-cell lung carcinoma; PMBCL, primary mediastinal B-cell lymphoma; RCC, renal cell carcinoma. Data source, FDA prescribing information.

The inhibitory co-receptor CTLA-4 is constitutively expressed by immunosuppressive regulatory T cells (Tregs), but it can be induced in T cells when activated by antigen-presenting cells (APC). In resting T cells, CTLA-4 is intracellularly localized in endosomes, but, upon T cell receptor (TCR) and CD28 costimulatory signaling activation, CTLA-4 translocates to the cell membrane (11). When exposed on cell surface, CTLA-4 prevents CD28 binding to B7.1 and B7.2 on APCs, thus precluding activation and proliferation of T cells (12). In fact, CTLA-4 inhibition causes a major immunostimulation, as experimentally shown in CTLA-4 knock-out (KO) mice, which die after few months due to uncontrolled lymphoproliferative disorders (13) and, in a clinical setting, by the reactivation of T cell-mediated tumor rejection.

Binding of PD-1 to its ligands, PD-L1 and programmed death-ligand-2 (PD-L2), also prevents T cell activation. PD-L1 is an inducible protein expressed in innate and adaptive immune cells, mesenchymal cells, and cancer cells (14), while PD-L2 is mainly expressed by APCs. PD-1 binding to PD-L1 significantly prevents immune responses directed against cancer cells, thereby altering T cell cytokine release, inhibiting TCR signaling, and abridging T cells/APCs interactions (15). The relevant role of this system in controlling T cell activity was demonstrated in PD-1-KO mice, which develop a spontaneous lupus-like disease caused by unrestrained autoreactive T cells (16). On the other hand, the inhibition of PD-1/PD-L1 binding in cancer can promote T cell activation and proliferation, ultimately leading to cytotoxicity in tumors.

MTC is reported to exhibit low PD-L1 expression in both tumor cells and tumor-infiltrating immune cells (17–19) and no microsatellite instability, irrespective of the presence/absence of either desmoplasia, lymph node metastases and/or RET mutation (18, 20).

However, PD-L1 positivity is associated with aggressive clinicopathological features (e.g., larger tumor size, lymph node or distant metastasis and higher TNM stage) (18, 19) and accounted as a predictor of structural recurrence and biochemical recurrence/persistent disease (19), and CTLA-4 expression is also correlated with advanced staging and structural recurrence-free survival (21), thus suggesting a possible prognostic role in the management of MTC (22).

## Aim

The aim of this review is to summarize and discuss available data on efficacy and safety of FDA-approved ICIs in patients with MTC.

## Materials and Methods

### Published Articles

We performed a literature search in the international online databases (PubMed, Web of Science, Scopus, and Embase) using the following terms: “immune checkpoint inhibitors”, CTLA-4, PD-L1, PD-1, atezolizumab, avelumab, cemiplimab, durvalumab, ipilimumab, nivolumab, pembrolizumab, “medullary thyroid cancer”, “medullary thyroid carcinoma”, “thyroid cancer”, “multiple endocrine neoplasia type 2”.

The search was last updated February 14, 2021.

### Registered Clinical Trials

By using the same keywords adopted for reviewing published articles, we conducted an in-depth search in the ClinicalTrials.gov registry, European Clinical Trials Database, and China Clinical Trials Register.

The search was last updated February 14, 2021.

## Results

### Published Articles

The initial literature search revealed a total of 108 published articles, two of which were pertinent to the study objectives.

Del Rivero et al. have recently reported the case of a 61-year-old male with recurrent MTC (23) showing sharp decline in serum calcitonin level while on avelumab. The patient had been successfully treated with off-label sunitinib for 5 years, but he was forced to withdraw the drug due to relevant side effects. He was then enrolled on a clinical trial with a yeast-based, CEA-targeted, therapeutic cancer vaccine (GI-6207) (24), and his calcitonin doubling time improved in 3 months. He then chose to have elective surgery to remove a neck lymph node and, per protocol, the vaccine was discontinued. Three months after surgery, his calcitonin level was still rising and he was enrolled on a phase I, open-label, multiple-ascending dose trial of avelumab (Avelumab in Metastatic or Locally Advanced Solid Tumors [JAVELIN Solid Tumor], NCT01772004). Thereafter, his calcitonin level decreased > 40% on 5 consecutive evaluations, and response assessment by RECIST v1.1 criteria (25) reported stable disease. However, an immune-related AE (i.e., asymptomatic grade 3 rise in lipase) ultimately led to mandatory treatment discontinuation. A subsequent analysis of a patient’s lymph node (resected post-vaccination) revealed that the tumor was PD-L1 positive.

Very preliminary results of a phase II trial evaluating nivolumab plus ipilimumab in patients with aggressive thyroid cancer (NCT03246958) are also available (26). Indeed, 7 patients with progressive MTC and prior TKI failure were included in an exploratory cohort of the study and assessed for radiographic response based on RECIST v1.1 criteria. Lack of partial response is reported for all the 7 patients, without giving further detail. Also, no safety information is provided for MTC as a single cohort (please see the *Registered Clinical Trials* section for more comprehensive description of the trial design).

### Registered Clinical Trials (RCTs)

We found 37 registered clinical trials (RCTs), five of which fully matched the aim of this review (**Table 2**).

**Table 2:** Registered clinical trials evaluating FDA-approved immune checkpoint inhibitors in medullary thyroid carcinoma.

ClinicalTrials.gov Identifier	Molecule	Trial name	Study phase	Medical condition under investigation	Assigned intervention	Primary outcome(s)	Estimated enrollment, n	Estimated study completion date	Trial status
NCT03753919	Durvalumab	A Phase II Study of Durvalumab (MEDI4736) Plus Tremelimumab for the Treatment of Patients With Progressive, Refractory Advanced Thyroid Carcinoma - The DUTHY Trial	Phase II	Metastatic thyroid cancer, including differentiated thyroid carcinoma, medullary thyroid carcinoma, and anaplastic thyroid cancer	Durvalumab 1500 mg plus tremelimumab 75 mg every 4 weeks up to 4 cycles followed by durvalumab 1500 mg every 4 weeks until disease progression, unacceptable toxicity or patients’ decision. Cohort 2 is composed by patients affected by advanced medullary thyroid carcinoma	Progression-free survival rate at 6 months [time frame: 6 months] according to RECIST 1.1 criteria Overall survival rate at 6 months [time frame: 6 months]	46	July 2021	Recruiting
NCT03246958	Nivolumab+ipilimumab	A Phase 2 Study of Nivolumab Plus Ipilimumab in RAI Refractory, Aggressive Thyroid Cancer With Exploratory Cohorts in Medullary and Anaplastic Thyroid Cancer	Phase II	Thyroid cancer (radioactive iodine-refractory, aggressive thyroid cancer with exploratory cohorts in medullary thyroid carcinoma and anaplastic thyroid cancer)	Arm I: ipilimumab will be administered *via* IV infusion, starting two weeks after nivolumab alone. Arm II: nivolumab will be administered *via* IV infusion, starting two weeks after ipilimumab alone	Radiographic response rate [time frame: 2 years], as determined by RECIST v1.1 (partial response+complete response)	53	March 2025	Active, not recruiting
NCT04514484	Nivolumab	Pilot Trial of Nivolumab Plus Cabozantinib for Advanced Solid Tumors in Patients With HIV Infection	Phase I	17 listed advanced, refractory, or metastatic solid tumors, including medullary thyroid carcinoma	Patients receive cabozantinib on days 1-28 and nivolumab on day 1. Cycles repeat every 28 days for up to 1 year or 1 year after a partial response is achieved, or 6 months after a complete response is achieved in the absence of disease progression or unacceptable toxicity	Incidence of dose limiting toxicities [time frame: 28 days], defined during cycle 1 of therapy	18	November 2025	Recruiting
NCT03072160	Pembrolizumab	Phase II Trial of Pembrolizumab in Recurrent or Metastatic Medullary Thyroid Cancer	Phase II	Medullary thyroid carcinoma	Pembrolizumab 200 mg will be administered as a 30 minute IV infusion every 3 weeks for two years	Determine whether a PD-1 inhibitor will permit a decline in calcitonin levels or response on imaging [time frame: one year]	17	November 2019	Completed (Results submitted)
NCT03012620	Pembrolizumab	Secured Access to Pembrolizumab for Patients With Selected Rare Cancer Types	Phase II	Sarcoma, ovarian neoplasm, central nervous system neoplasm, thyroid neoplasm (including medullary thyroid carcinoma), neuroendocrine carcinoma, germ cell and embryonal neoplasms, NK/T-cell lymphoma	Pembrolizumab 200 mg on day 1 of every 21 day cycle	Objective response rate [time frame: measured at the first scheduled disease assessment following study treatment initiation (day 84 ± 7 days)] according to RECIST v1.1	350	December 2023	Recruiting

NCT03753919 (A Phase II Study of Durvalumab (MEDI4736) Plus Tremelimumab for the Treatment of Patients With Progressive, Refractory Advanced Thyroid Carcinoma - The DUTHY Trial) is a prospective, multi-center, open label, stratified, exploratory phase II study whose aim is to evaluate the following outcomes in patients affected by advanced thyroid cancer (estimated enrollment: 46 patients). Primary outcomes are PFS rate at 6 months and overall survival (OS) rate at 6 months; secondary outcomes comprise overall response rate (ORR), duration of response (DoR), median PFS, incidence of treatment-emergent AEs, median OS, and response status after start of study treatment. According to the primary histotype, patients are divided in three cohorts: i) advanced, radioiodine-refractory differentiated thyroid carcinoma (DTC), including papillary, follicular, Hürtle cell and poorly-differentiated thyroid carcinoma (Cohort 1); ii) advanced MTC (Cohort 2); iii) anaplastic thyroid cancer (ATC) (Cohort 3). Each cohort is planned to receive durvalumab plus tremelimumab (anti-CTLA-4 antibody, not yet approved by FDA) every 4 weeks up to 4 cycles followed by durvalumab alone every 4 weeks until progression, unacceptable toxicity or withdrawal. The study started in April 2019, with the estimated study completion date being July 2021. The present study status is “Recruiting”.

NCT03246958 (A Phase 2 Study of Nivolumab Plus Ipilimumab in RAI Refractory, Aggressive Thyroid Cancer With Exploratory Cohorts in Medullary and Anaplastic Thyroid Cancer) is a phase II clinical trial evaluating nivolumab in combination with ipilimumab, as a possible treatment for thyroid cancer, focusing on effectiveness (estimated enrollment: 53 patients). The primary endpoint is radiographic response rate as determined by RECIST v1.1 (i.e. partial response plus complete response), whereas secondary outcomes are PFS, OS, and tolerability at two years. This trial is designed to recruit patients with metastatic, progressive, RAI refractory DTC with exploratory cohorts in ATC (7 patients), and incurable, progressive MTC with prior TKI failure (10 patients). Participants aged ≥18 years are divided in two experimental arms: the first arm will be administered nivolumab alone for two weeks followed by nivolumab plus ipilimumab, whereas the second arm ipilimumab alone for two weeks followed by nivolumab/ipilimumab combination therapy. The study started in September 2017. The estimated study completion date is set for March 2025. The present study status is classified as “Active, not recruiting”. As above reported, very preliminary results of this trial have been recently published recently (26).

NCT04514484 (Pilot Trial of Nivolumab Plus Cabozantinib for Advanced Solid Tumors in Patients With HIV Infection) is a phase I trial that aims at defining in HIV-positive patients with advanced/metastatic solid cancer (estimated enrollment: 18 patients) the incidence of dose limiting toxicities during cycle 1 of therapy with cabozantinib and nivolumab (primary outcome). Secondary outcomes include the assessment of immune status (CD4 and CD8 cell counts) at each time point from baseline, HIV viral loads, changes in serum markers of immune activation, in immune checkpoint markers, in angiogenesis markers, and in infiltrating immune cell markers. According to the protocol, patients ≥18 years old receive cabozantinib on days 1-28 and nivolumab on day 1. Cycles repeat every 28 days for up to 1 year or 1 year after a partial response is achieved, or 6 months after a complete response is achieved in the absence of disease progression or unacceptable toxicity. The study started in November 2020. The estimated study completion date is November 2025. The present study status is “recruiting”.

NCT03072160 (Phase II Trial of Pembrolizumab in Recurrent or Metastatic Medullary Thyroid Cancer) is a phase II, open label, single center clinical trial aimed to determine, in patients having or not having undergone previous vaccine therapy (estimated enrollment: 15 patients in each cohort), whether a PD-1 inhibitor may allow for a decline in calcitonin levels or radiographic response (primary outcome); secondary outcomes include impact of previous therapeutic cancer vaccine on response rates, evaluation of immune responses in each cohort, changes in CEA and calcitonin kinetics, PFS, OS and safety. All patients will receive pembrolizumab 200 mg every 3 weeks. The study started in June 2017 and was completed in November 2019, and indeed the present study status is “completed”. On 11 February 2021, very preliminary results appeared in the Study Results section of the ClinicalTrials.gov registry. Thirteen patients were enrolled in the cancer vaccine arm (2/13 patients completed the trial), and 4 patients were enrolled in the control arm (none completed the trial). Disease progression was observed in 1/13 patients of the first arm, and in 1/4 patients of the second arm.

NCT03012620 (Secured Access to Pembrolizumab for Patients With Selected Rare Cancer Types) is a phase II, 2, non-randomized, open-label, multicenter study which aims to investigate the efficacy and safety of pembrolizumab in 7 different cohorts of patients with unresectable/locally advanced/metastatic rare cancers for which no other treatment options are available (estimated enrollment: 350 patients). Primary outcome is ORR, whereas secondary outcomes comprise PFS, OS, DoR, time to response, frequency and severity of AEs, and ORR/PFS/OS in subgroups of subjects with high versus low expression of PD-L1, CD4, FOX3 and other immune markers. According to the protocol, cohort 4 features rare thyroid cancer patients of ≥18 years, including MTC; these patients, same as for all other cohorts, are planned to receive pembrolizumab 200 mg on day 1 of every 21-day cycle. The study started in July 2017 and its estimated completion date is December 2023. The present study status is “recruiting”.

## Discussion

Our review shows, despite very limited published evidence, an increasing attention to the possibility of treating MTC with ICIs, and indeed we found 5 ongoing RCTs with FDA-approved drugs that collectively involve nearly 500 patients with solid tumors, including MTC.

As an additional sign of interest, two trials investigating camrelizumab, a novel PD-1 inhibitor recently approved in China for the treatment of relapsed/refractory classical Hodgkin lymphoma (27, 28), are also intended to recruit patients with MTC, i.e. the NCT04612894 (The Efficacy and Safety of Anti-PD-1 Antibody Camrelizumab Combined With Apatinib for Neoadjuvant Therapy in Locally Advanced Thyroid Cancer: a Phase II Study), and NCT04521348 (A Phase II Study to Explore the Safety and Efficacy of Multiple Target Kinase Inhibitor (mTKI) Combined With Anti-Programmed Death-1(PD-1) Antibody in the Treatment of Advanced Thyroid Cancer) trials.

Notably, PD-1 and CTLA-4 have non-redundant immunosuppressive effects, paving the way for the development of clinical protocols with antibodies targeting the two pathways (29). Combination therapy with anti-PD-1 and anti-CTLA-4 drugs (durvalumab plus tremelimumab, NCT03753919 trial; nivolumab plus ipilimumab, NCT03246958 trial) is giving rise to great expectations in MTC. Overall, there is reliable evidence supporting a greater efficacy of the combined PD-1/CTLA-4 blockade over the two monotherapies in reversing tumor immune inhibition (30–32).

A number of different new scenarios could be opened by combinations or sequential schemes with other anti-tumor treatment modalities.

Systemic chemotherapy has been proposed to exert synergistic effects when combined with PD-1/PD-L1 blocking drugs in non-small-cell lung carcinoma (33). Indeed, chemotherapeutic agents may affect antitumor immunity both indirectly stimulating the immune system through immunogenic death of tumor cells, and directly regulating immune cell subsets, thereby reducing immunosuppression in the tumor microenvironment (TME) (34).

Second- or third-line treatment with ICIs has become increasingly common for patients with advanced disease who have already received other types of anticancer therapies (35).

It has been hypothesized that previous administration of cancer vaccines can drive immune cells to the TME and upregulate PD-L1 expression in the tumor cells due to cytokine release in the TME, thus giving a chance for anti-PD-L1/PD-1 drugs in patients who may not have otherwise benefited from such immunotherapies (36, 37). Interestingly, in the above-mentioned case report by Del Rivero et al. (23), the 61-year-old male showing >40% decrease in calcitonin level while on avelumab had previously undergone a 3-month trial with the GI-6207 cancer vaccine. Although a subsequent analysis of a patient’s lymph node (resected post-vaccination) revealed that the tumor was PD-L1 positive, no information about PD-L1 status before vaccination is available. As a further complication in this case’s assessment, the patient had been previously treated with the TKI sunitinib, which is acknowledged to deplete Tregs, and may have affected PD-L1 status as well (38).

The therapeutic potential of FDA-approved atezolizumab, avelumab, ipilimumab and pembrolizumab has also been investigated in NENs other than MTC (39, 40), thereby confirming a strong interest for ICI therapy in this subset of tumors.

## Conclusion

Despite the lack of evidence regarding the use of ICIs in MTC, it should be considered that all the aforementioned RCTs saw first light in the last three years, thus indicating a growing interest of researchers in this field. Results coming from these trials, and hopefully from additional ones in the next future, will help clarify whether these drugs may represent a new weapon in favor of patients with MTC, and determine their position in the treatment algorithm.

## Author Contributions

SDM, AD, GF, TFl, and TFe were responsible for the design, the methodology, the draft preparation, the reviewing and editing. AC and AF were responsible for the supervision. All authors contributed to the article and approved the submitted version.

## Funding

This work was supported by the Italian Ministry of Education, University and Research (MIUR): PRIN 2017Z3N3YC.

## Conflict of Interest

The authors declare that the research was conducted in the absence of any commercial or financial relationships that could be construed as a potential conflict of interest.

## Abbreviations

AEs, adverse events; APCs, antigen-presenting cells; ATC, anaplastic thyroid cancer; DTC, differentiated thyroid carcinoma; CTLA-4, cytotoxic T-lymphocyte-associated protein 4; DoR, duration of response; FDA, Food and Drug Administration; ICIs, immune checkpoints inhibitors; KO, knock-out; MTC, medullary thyroid carcinoma; NEN, neuroendocrine neoplasm; ORR, overall response rate; OS, overall survival; PD-1, programmed cell death protein-1; PD-L1, programmed death-ligand-1; PD-L2, programmed death-ligand-2; PFS, progression-free survival; RCTs, registered clinical trials; RET, rearranged during transfection; TME, tumor microenvironment; TCR, T cell receptor; TKIs, tyrosine kinase inhibitors; Tregs, regulatory T cells.
